# Beyond physical health: the role of psychosocial challenges and stigma in tackling the COVID-19 pandemic—A scoping review

**DOI:** 10.3389/fpsyt.2023.1180252

**Published:** 2023-07-11

**Authors:** Lakshit Jain, Siddhi Bhivandkar, Huma Baqir, Sheikh Shoib, Nirav Nimavat, Anmol Mohan, Aarij Shakil Zubair, Muhammad Youshay Jawad, Nazar Muhammed, Rizwan Ahmed, Vishi Sachdeva, Saeed Ahmed

**Affiliations:** ^1^Connecticut Valley Hospital, Middletown, CT, United States; ^2^Department of Psychiatry, University of Connecticut, Farmington, CT, United States; ^3^Department of Psychiatry, St. Elizabeth’s Medical Center, Boston University, Boston, MA, United States; ^4^Department of Psychiatry, University at Buffalo, Buffalo, NY, United States; ^5^Department of Psychiatry, Jawahar Lal Nehru Memorial Hospital, Srinagar, India; ^6^Dr. Kiran C. Patel Medical College and Research Institute, Bharuch, India; ^7^Department of Medicine, Karachi Medical and Dental College, Karachi, Pakistan; ^8^Touro College of Osteopathic Medicine, New York, NY, United States; ^9^King Edward Medical University, Lahore, Pakistan; ^10^Department of Psychiatry, Cornerstone Family Healthcare, New York, NY, United States; ^11^Liaquat College of Medicine and Dentistry, Karachi, Pakistan; ^12^Adesh Institute of Medical Sciences and Research, Bathinda, India; ^13^Addiction Psychiatry, Rutland Regional Medical Center, Rutland, VT, United States

**Keywords:** COVID-19, pandemic, psychocultural, psychosocial, stigma, mental health, conspiracy, infodemic

## Abstract

**Background:**

The socio-cultural response to the Coronavirus Disease 2019 (COVID-19) and the level of adherence to evidence-based guidelines played a crucial role in determining the morbidity and mortality outcomes during the pandemic. This review aims to evaluate the impact of stigma and psycho-socio-cultural challenges on efforts to control the COVID-19 pandemic and to identify ways to mitigate such challenges in future pandemics.

**Methods:**

Using keywords including COVID-19, coronavirus, stigma, psychosocial challenges, and others, the authors searched seven major databases with a time limitation of July 2021, which yielded 2,038 results. Out of these, 15 papers were included in this review.

**Results:**

The findings of the review indicated that several psychosocial, socio-economic, and ethno-cultural factors are linked to the transmission and control of COVID-19. The research revealed that stigma and related psychosocial challenges and others, such as anxiety, fear, and stigma-driven social isolation, have resulted in significant mental health problems.

**Discussion:**

The review underscores the negative impact of stigma on COVID-19 patients, survivors, and the general population. Addressing stigma and psychosocial challenges is crucial to effectively manage the current pandemic and to prevent similar challenges during future public health crises.

## 1. Introduction

The COVID-19 pandemic has triggered varying responses from cultures and nations across the world. The emergence of the novel SARS-CoV-2 virus led to the dissemination of information through various media channels in an effort to control its spread. However, the promotion of awareness was accompanied by a significant amount of false or misleading information related to COVID-19, which spread rapidly and had a negative impact on our ability to effectively manage the pandemic. This phenomenon, referred to as an “infodemic” (1), contributed to the development of stigma. Stigma, defined as an attribute that links a person to an undesirable stereotype (2), results in social labeling that hinders full acceptance by society and leads to discrimination, elevated individual stress, and healthcare disparities (2). The instinctual fear response to stigma can foster biases and discriminatory behaviors, particularly when coupled with a lack of knowledge. Despite efforts to mitigate its spread, false and unscientific information about COVID-19 and conspiracy theories continue to persist on the internet (2). The COVID-19 pandemic has led to the dissemination of false information and conspiracy theories through various media channels, which has contributed to stigma associated with the disease. The false information and conspiracy theories, such as the belief that 5G technology is responsible for the spread of COVID-19 (3), have spread quickly and led to significant healthcare disparities (4, 5). A survey conducted in 2020 found that 36% of individuals believed that the pandemic was planned when told so as part of the experiment (5). These conspiracy theories not only contribute to the spread of the virus but also cause significant stigma. To flatten the curve of positive cases and deaths from SARS-CoV-2, governments across the world implemented various isolating measures such as stay-at-home orders, social distancing, quarantining potentially infected individuals, face coverings, shutting down non-essential businesses and social ceremonies at varying levels of strictness. However, the strength of a culture’s response to an outbreak and willingness of the people to comply with the public health officials played a significant role in morbidity and mortality outcomes (6). Despite national and global health leaders’ insistence on these isolating measures, psychosocial cultural phenomena—stigma, conspiracy theories, individualism, and political agenda—remained obstacles to the containment of COVID-19. Disregarding the nuances of these obstacles and letting science hold complete sway could alienate the cultures and the people. This may compound barriers to managing the pandemic.

Ultimately, the healthiest horizon for all requires a compromise between the medical and the psychosocial-cultural sectors (7). Insufficient knowledge and awareness about the transmission, treatment, and prevention of SARS-CoV-2 can contribute to increased stigma in communities. To mitigate the impact of stigma, effective strategies can be implemented through social media to reduce fear and provide accurate and timely information about the high-risk groups, preventive measures, and treatment modalities. This scoping review aims to analyze the issues related to stigma and psychosocial challenges that have emerged during the COVID-19 pandemic, and to provide recommendations for authorities and healthcare professionals to address them in preparation for future pandemics.

## 2. Materials and methods

**FIGURE 1:**
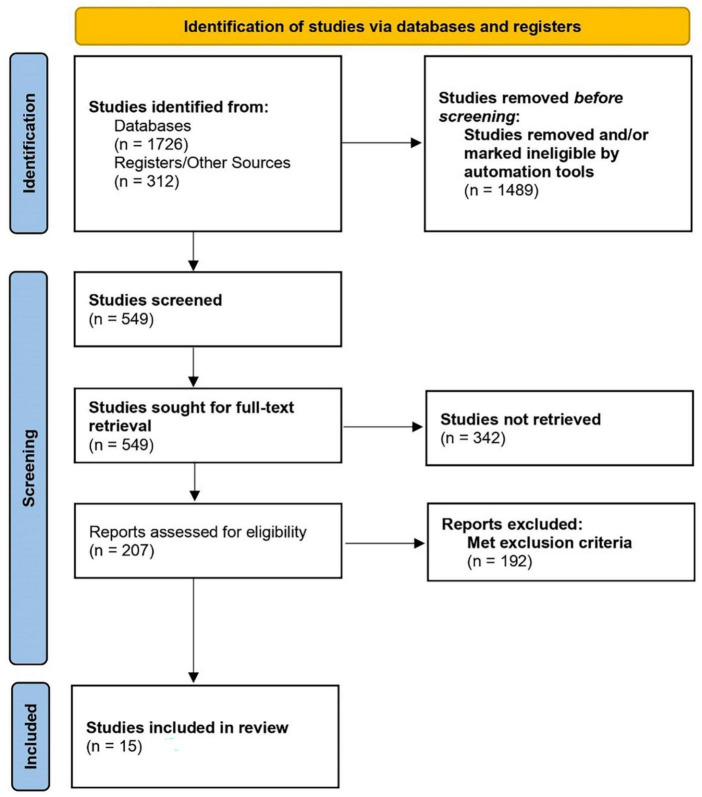
PRISMA flow diagram.

A literature search was performed following PRISMA guidelines using the following databases/registers: PubMed, Embase, Litcovid, bioRxiv, medRxiv, Web of Science, and PsychINFO, from January 2020 to July 2021. Gray literature was searched through a web search and Google Scholar. We used combinations of the following keywords applying BOOLEAN logic (AND/OR): “COVID-19, coronavirus, SARS-CoV-2”, “COVID- 19 pandemic” “Covid” AND “Stigma” OR “psychosocial challenges” OR “mental health” OR “cultural issues” OR “cultural challenges” OR “stigma” OR “mental health access”. The initial search was performed by authors RA and SB through these databases, which generated 2,038 reports. After excluding all duplicates and completely off-topic titles, 549 citations were left. We screened records for inclusion criteria and excluded 342 papers. The remaining 207 publications were manually screened by three authors (SS, HB, VS), any disagreements were mediated by the first author (SA). Studies were removed if they were commentaries, case reports, case series, opinions, workshops, unpublished data, and reviews. A total of 192 papers did not fit the inclusion criteria, resulting in 15 full-text articles that met the inclusion criteria (Figure 1). The included 15 studies are summarized in Table 1.

**TABLE 1:** Summary of included references reporting stigma and psychosocial challenges of COVID-19.

No.	References	Country	Study design	Title of the study	Sample size	Objectives	Results
1	Herawati et al. (10)	Indonesia	Cross-sectional	Stigma, anxiety, and religiosity on COVID-19 preventative measures	451	To determine the effect of stigma, level of anxiety, level of religiosity, and economic condition on COVID-19 preventive efforts among college students	1. Most dominant influential variable on COVID-19 preventive efforts was *stigma* with OR of 2.256. (*p* = 0.000); 2. *Level of religiosity* had no association with COVID-19 preventive measures (*p* = 0.174); 3. Apart from stigma, *anxiety* (*p* = 0.013) and *economic condition* (0.031) also had significant impact on preventive measures
2	Ugidos et al. (40)	Spain	Longitudinal	Evolution of intersectional perceived discrimination and internalized stigma during COVID-19 lockdown among the general population in Spain	1st survey: 3,489; 2nd survey: 1,041; 3rd survey: 568	To analyze, longitudinally, the evolution of intersectional perceived discrimination and internalized stigma among the general population of Spain, at three points in time throughout the confinement.	1. From the first to the second evaluation, results show a significant increase in intersectional discrimination and internalized *stigma* (*p* < 0.001); 2. The trends found show that *discrimination and internalized stigma* increase with the evolution of the crisis, decrease with the beginning of recovery and return to normal, although without returning to previous levels
3	Shokri et al. (41)	Iran	Cross sectional	Stigma and COVID-19 in Iran: A rapid assessment	1,000	To investigate the perceived stigma among Iranians following the COVID-19 pandemic	1. Entirely, 99% of people predicted at least one *stigma-endorsing response* and the mean perceived stigma related COVID-19 was 5.50 (IQR: 3.75–6.87) of 10-point scale; 2. The mean stigma sub-scores were highest for perceived external stigma 6.73 (IQR: 5–8.75) followed by *disclosure stigma* 4.95 (IQR: 0–10); 3. Self-employers were more concerned about *disclosing their illness* than those with governmental jobs (2563.93 vs. 4.3164.14, *P* < 0.05), and also had an *overall higher stigma score*; 5.72 vs. 5.19, *P* < 0.05
4	Amir (16)	Uganda	Cross-sectional exploratory	COVID-19 and its related stigma: A qualitative study among survivors in Kampala, Uganda.	30 (COVID-19 Survivors)	To explore COVID-19 related stigma among survivors in Kampala, Uganda using in depth interviews	The results revealed that a common form of stigma among survivors was *social rejection* followed by *labeling*
5	Miconi et al. (17)	Canada	Cross-sectional	Ethno-cultural disparities in mental health during the COVID-19 pandemic: a cross-sectional study on the impact of exposure to the virus and COVID-19-related discrimination and stigma on mental health across ethno-cultural groups in Quebec (Canada)	3,273	To investigate the association of exposure to the virus, COVID-19-related discrimination and stigma with mental health during the COVID-19 pandemic, in a culturally diverse sample of adults in Quebec (Canada).	1. *Exposure* to the virus, COVID-19-related *discrimination*, and *stigma* were associated with poorer mental health; 2. Mental health varied significantly based on *socioeconomic status* and *ethno-cultural group*, with those with *lower incomes* and Arab participants reporting higher *psychological distress*; 3. Associations with mental health varied across ethno-cultural groups, with exposed and discriminated Black participants reporting higher *mental distress*
6	Yuan et al. (12)	China	Case-control	COVID-19-related stigma and its sociodemographic correlates: a comparative study	154 COVID survivors and 194 healthy controls	To compare differences in stigma experiences of COVID-19 survivors vs. healthy controls after the COVID-19 outbreak peak in China	1. Compared with healthy controls, COVID-19 survivors reported more overall *stigma* (*p* < 0.001), and stigma in domains of *social rejection* (*p* < 0.001), *financial insecurity* (*p* < 0.001), *internalized shame* (*p* < 0.001), and *social isolation* (*p* < 0.001); 2. *Status* as a COVID-19 survivor, having family members infected with COVID-19, being married, *economic* loss during the COVID-19 pandemic, and *depressive symptoms* were positively associated with higher overall stigma levels (all *p* values < 0.05).
7	Lohiniva et al. (8)	Finland	Cross sectional based on one to one interviews	Learning about COVID-19-related stigma, quarantine and isolation experiences in Finland	64	To review the forms, drivers, outcomes, and impact of social stigma toward those with corona virus and COVID-19 and their family members, and to shed light on their quarantine experience	1. Respondents did not feel a *sense of closure* after their isolation and quarantine had ended because of perceived *stigma and self-stigma*, and worry that they could still infect people around them; 2. Stigma resulted in *delayed health seeking behavior* among symptomatic patients including testing which can speed the transmission of the virus rapidly; 3. Health officials left out a number of important audiences in communications about the virus: (i) those who cared for sick (caretakers) for critical information and opportunities to discuss and get advice, (ii) children and teenagers did not have a specific channel to communicate with health officials to gain information or share fears and concerns, (iii) asymptomatic household members or those who tested negative received less attention although they seemed to have equally pressing uncertainties
8	Tomczyk et al. (11)	Germany	Online survey	Social distancing and stigma: association between compliance with behavioral recommendations, risk perception, and stigmatizing attitudes during the COVID-19 outbreak	157	To examine patterns of intentions to comply with behavioral recommendations to contain the COVID-19 pandemic in the German population *via* latent class analysis.; tonspect the role of stigma in non-compliance while considering sociodemographic differences, risk perception, and knowledge of adaptive behaviors; to explore intercultural similarities and differences of compliance by focusing on the German population, whereas previous research mostly focused on Asian populations	It discussed the positive association between public stigma and compliance. Compared to high compliance, low compliance was associated with male gender, young age and lower public stigma.
9	Jiang et al. (4)	China	Cross sectional	COVID-19-related stigma and its’ influencing factors: a rapid nationwide study in China	5,039	(1) To evaluate the prevalence of stigma during the COVID-19 outbreak in China (2) To assess the association of stigma, health literacy, and sociodemographic characteristics during the COVID-19 epidemic.	1. People aged over 40, lived in areas with severe epidemics (aOR = 2.15, 95% CI [1.12–4.13]), and who felt it difficult to find and understand information about COVID-19 (aOR = 1.91, 95% CI [1.08–3.27]; aOR = 1.88, 95% CI [1.08–3.29]) were more likely to *stigmatize* COVID-19 patients; 2. People who were male, aged 41–50, and had *difficulty understanding* information (aOR = 2.08, 95% CI [1.17–3.69]) were more likely to stigmatize people from Wuhan
10	Mahmoudi et al. (19)	Iran	Cross-sectional	A mediating role for mental health in associations between COVID-19-related self-stigma, PTSD, quality of life, and insomnia among patients recovered from COVID-19	844 (recovered)	To investigate whether poor mental health may mediate concerns related to infection with COVID-19 (i.e., self- stigma and PTSD) and outcomes such as poor sleep and HRQoL in people having recently recovered from COVID-19	1. *Insomnia, PTSD, and COVID-19-related self-stigma* displayed significant direct associations (*r* = 0.334–0.454; *p* < 0.01); 2. Mental health may mediate effects of COVID-19-related *self-stigma and PTSD on quality of life and insomnia*
11	Earnshaw et al. (9)	USA	Online survey	Anticipated stigma, stereotypes, and COVID-19 testing.	845	To explore whether anticipated stigma and stereotypes are associated with likelihood of COVID-19 testing. Knowledge and fear of COVID-19 were included as control variables in analyses	1. Participants who anticipated greater COVID-19 stigma and endorsed COVID-19 stereotypes to a greater degree reported that they would be *less likely to seek a COVID-19 test* (*p* < 0.001); 2. Participants with *greater COVID-19 knowledge and fear* reported that they would be more likely to seek a COVID-19 test. Participant *sociodemographic variables* were not associated with reported likelihood of testing. The adjusted *R*^2^ for the model was 0.26 (SE 0.80, *p* < 0.001)
12	Kang et al. (18)	Korea	Retrospective analysis of medical records	The psychological burden of COVID-19 stigma: evaluation of the mental health of isolated mild condition COVID-19 patients	107	To assess the mental health issues (anxiety, depression, PTSD, and somatic symptoms) of the mild condition coronavirus disease 2019 (COVID-19) patients admitted to a community treatment center (CTC) in Korea; to examine the relationship of COVID-19 stigma with psychiatric conditions	1. For *depression and anxiety*, previous psychiatric history and stigma of COVID-19 infection were significant risk factors; 2. For *PTSD, previous psychiatric history and stigma of COVID-19 infection as well as total duration of isolation* were found to be significant risk factors
13	Singh et al. (13)	India	Telephonic survey	Health, psychosocial, and economic impacts of the COVID-19 pandemic on people with chronic conditions in India: a mixed methods study	2,335	To assess the health, psychosocial and economic impacts of the COVID-19 pandemic on people with chronic conditions in India	During the COVID-19 lockdowns in India, 83% of participants reported difficulty in *accessing healthcare*, 17% faced difficulties in *accessing medicines*, 59% reported *loss of income*, 38% *lost jobs*, and 28% *reduced fruit and vegetable consumption*
14	Bodrud-Doza et al. (14)	Bangladesh	Perception based online questionnaire	Psychosocial and socio-economic crisis in Bangladesh due to COVID-19 pandemic: a perception-based assessment	1,066	To analyze the psychosocial, socio-economic, and possible environmental crisis based on public perception in Bangladesh due to the COVID-19 outbreak	1. There was a negative association between the fragile health system of Bangladesh and the government’s ability to deal with the pandemic (*p* < 0.05), revealing the *poor governance in the healthcare system*; 2. A positive association of shutdown and social distancing with the fear of losing one’s own or a family members’ life, influenced by a *lack of healthcare treatment* (*p* < 0.05), reveals that, due to the decision of shutting down normal activities, people may be experiencing mental and economic stress; 3. Positive association of the *socio-economic impact* of the shutdown with poor people’s suffering, the price hike of basic essentials, the hindering of formal education (*p* < 0.05), and the possibility of a severe socio-economic and health crisis being aggravated
15	Zakar et al. (15)	Pakistan	Cross-sectional	Socio-cultural challenges in the implementation of COVID-19 public health measures: Results from a qualitative study in Punjab, Pakistan	34	To explore the social and behavioral response to COVID-19 and unveils challenges in the implementation of related public health measures in Pakistan	Lockdown strategy impacting *income* of the population. Adherence to social distancing measures dependent on living conditions. *Misleading information* on COVID-19

**TABLE 2:** Quality assessment, sample selection, measurement scales, analysis, and interpretation of findings.

Identifiers and quality assessment			Sample selection		Measurements and analysis		Results and discussion	
References	Number	Grade	Selection of participants	Retaining participants/ Compensation	Measurements	Statistical analysis	Interpretation	Discussion
Herawati et al. (10)	1	Medium	One exclusion criterion was age > 30 years old, however, the table of characteristics of study subjects reported range of 17–49 years. The Snowball Sampling technique is inappropriate, since college student should be easily contactable by their school, therefore voiding the justification of them being “hard to reach population”	Not applicable	The use of Hamilton Anxiety Rating Scale is widely accepted, and the authors mentioned that several local studies have used them.	The use of logistic regression is acceptable, given the binary outcome of interest	The authors seem to interpreted Odds Ratio as Risk, which is problematic. The “event” is not rare, therefore the OR will not estimate RR	Supported by results, however, the comparison to China is quite far-fetched, since the majors are different, and the tools used to measure anxiety are not HARS
Ugidos et al. (40)	2	High	Recruitments were based on existing database of students and workers, therefore the elderly are less represented. But this was discussed and acknowledged by the authors (i.e., due to impracticalities in recruiting elderly using offline forms)	Significant loss of participants at subsequent time points, due to “loss of interest.” The authors did not seem to foresaw this and made some efforts to maintain retention (i.e., through incentives or supports)	The questionnaires used were tailored to match the objectives, which is appropriate → the InDI-D used “Presence of COVID-19” as the condition	The use of Linear Mixed Model is acceptable. The authors did not perform imputation due to the missingness properties not known (quite possible MNAR, Missing Not At Random)	In line with the reported models	It seems that the questionnaires used provide acceptable fit, the Pseudo-R squared (Tables 2, 3) are quite good, given the number of items and the three time points. These supported the interpretation of the models.
Shokri et al. (41)	3	Medium	There seems to be unclear procedures on participants recruitment. “the questionnaire was shared to participants through email, Instagram direct, WhatsApp direct, and Telegram groups.” Did they stop as soon as the recruited participants reached 1,000? Were the methods carried out in parallel?	Not applicable	The Berger HIV stigma scale, modified for Ebola was used. However, the concept of internalized stigma of Berger’s and COVID-19 is very distinct. This implication was not discussed.	The use of ANOVA and *T*-test are acceptable. The significance level was set at 0.005, but subsequently at 0.05 in the results (most likely a typo in the method section)	Straight-forward and concise interpretation.	Supported by the data, particularly the context of governmental jobs and self-employed situation were discussed.
Amir (16)	4	Low	It is highly disturbing that “saturation principle” was used to stop the recruitments, but this was based on a single interpreter/coder, without cross examinations. Thus whether saturation was truly achieved is questionable.	Not applicable	Seven-phase data analysis framework was used but all identifications of themes relied on this single author → very prone to coding bias	Pure qualitative study, without statistical analysis	All of the themes are supported by actual quotes by the participants	Supported by the interviews
Miconi et al. (17)	5	Medium	Based on Leger Opinion Panel. Therefore some degree of selection bias is expected (non-participants of Panel would not be able to participate)	The authors’ decision to reward participants based on survey completion time was highly questionable (CAD 0.5–2). This practice has been discouraged by survey guidelines.	The use of Hopkins Symptom Checklist-10 is acceptable, however, to my knowledge it has not been validated in Arab population (which contributes *n* = 450 in this study)	The use of factor analysis to dimensionally reduce the data complexities of HSCL-10 is acceptable	Supported by the results	The discussion draws comparison to the UK and US which investigated COVID-19 in multi-racial settings. The recommendations (focusing on ethnic minorities) are supported by the findings in the UK and US
Yuan et al. (12)	6	High	COVID-19 survivors were compared with healthy control of the same city (by convenience sampling).	Not applicable	Fatigue and Stigma were assessed using PHQ-9 and Social Impact Scale (a generic stigma scale). The SIS has been validated in Chinese population previously, but not for COVID-19	*T*-test, ANOVA, and generalized linear model were used, which are appropriate.	The interpretation were based on the comparison between survivors and healthy controls, appropriate given the study design’s limitation of convenience sampling.	The recommendations were based on the findings, however, the decision to not performed matched controls was not explained. Almost half of the survivors were male, but only 20.6% were male in the control group. This imbalance might skew the findings toward the null
Lohiniva et al. (8)	7	High	Based on maximum variation and data saturation principles, which are standard practices for family-based interviews	Not applicable	Framework analysis following the Health Stigma Framework. Four investigators discussed the coding and framework analysis	Pure qualitative study, without statistical analysis	All of the themes are supported by actual quotes by the participants. Attributions of the quotes were presented consistently	The themes and recommendation being discussed were based on the interview results
Tomczyk et al. (11)	8	High	Based on online advertisements through Facebook.	Participants received EUR 5 if they finished the set of questions	Mostly based on mental health questionnaires. But some of the adaptation was quite questionable, for example the “Persons with COVID-19 should not be allowed to have a driver’s license” question	Since the classes were 3, producing three pairs of 1-2, 2-3, and 1-3, the use of Multinomial Logistic Regression is appropriate.	In line with the reported results	The tailored health promotion efforts targeting youths was supported by the RRR results
Jiang et al. (4)	9	High	The study has a specific objective of exploring stigma of Chinese people toward people from Wuhan, therefore the recruitment method is acceptable	Not applicable	Adaptation of stigma related to tuberculosis was used. Pre-testing was performed to ensure appropriateness to COVID-19	Chi-square and logistic regression were used, which are appropriate.	In line with the reported results	There are several inconsistencies in the discussion section. The authors acknowledged that the sampling was not representative and not probabilistic, but they presented and discussed the findings as prevalence. This is clearly inappropriate.
Mahmoudi et al. (19)	10	Medium	Convenience sampling method is deemed acceptable, since they objective of the study was to have PCR test and Chest CT. One minor concern is why the four hospitals were used instead of the others was not properly explained.	Not applicable	PTSD and Self-Stigma Scale-Short (originally was designed for mental health, immigrant, and sexual-orientation minority groups) were used. Adaptation for COVID-19 was performed. Insomnia and MHI-5 were also used	Since the classes were 3, producing three pairs of 1-2, 2-3, and 1-3, the use of Multinomial Logistic Regression is appropriate.	The authors seem to be a bit over optimistic in reporting the goodness of fit. The root mean square error of approximation (RMSEA) value indicated close to mediocre fit, not “satisfactory” as the authors have implied	The mediation analysis were quite acceptable, however, the discussion did not acknowledge that the fit was rather mediocre → some unexplored and unmeasured factors might be necessary to be investigated to improve the current mediation analysis
Earnshaw et al. (9)	11	Low	Based on Amazon MTurk Panel. Therefore some degree of selection bias is expected (non-participants of MTurk would not be able to participate)	Participants received USD 2 if they finished the set of questions	Chronic Illness Anticipated Stigma Scale was used, but only relevant items for COVID-19 were retained	Linear regression was used for the analysis	The decision to display testing likelihood without confounder adjustment is questionable. Gender and Race (two covariates which were collected in the study) should be included in the analysis.	The interpretation is greatly limited by potential confounders not considered by the authors.
Kang et al. (18)	12	High	Retrospective analysis of medical record of a community isolation facility	All of the patients analyzed. Time cut-off were used to exclude participants.	The use of MERS questionnaire was appropriate, given the relative similarity of transmission methods between MERS and COVID-19 viruses.	Acceptable. Limited number of participants complicate further analysis of the data. For example, only *n* = 32 have been isolated up to the 4th week. Therefore the absence of further analysis is understandable	In line with the reported results	The authors promoted the CTC model in handling COVID. Notably the comparison to other models were given with careful consideration that they are in other countries with different system and different diseases (H1N1)
Singh et al. (13)	13	High	Participants were cohort participants of CARRS and India-UDAY. Re-contacted to participate in the current research.	No information was given on the approach to recruit participants, and whether refusal translate to exclusion from cohorts future investigations (which may dis-incentivize participants)	The anxiety was assessed by generalized anxiety disorder questionnaire, an established method. The remaining items are specifically developed by the authors for this study (without adequate validation or pre-testing)	For the qualitative part the authors used “illustrative non-attributable quotations,” without any justification. The data collection protocol recruited a diverse set of participants (i.e., not a very specific group of people), therefore attribution should be performed.	Correct interpretation of the Odds Ratios and factors that changes the OR (with specific examples for ease of interpretation)	The tailored health promotion efforts targeting youths was supported by the RRR results
Bodrud-Doza et al. (14)	14	Medium	Email and social platform recruitments (Facebook, WhatsApp, etc.) combined with targeted database of hard to reach group.	Not applicable	The questions were specifically developed for COVID-19 instead of adapting them from previous questionnaires. Expert consultation was used to validate the questions	There were 46 items which the authors attempted to be reduced to a manageable levels of number of variables for easier interpretation. The use of CTT PCA sequentially followed by CA were unusual, but not unheard of.	There is one major concern regarding the Scree Plot and Eigen-cutoff. The alternative of PC1-PC5 seems to be more appropriate, instead of including nine PCs (**Figure 2** and **Table 3**)	The authors provide additional information and suggestion which were reasonable, but not supported by the data, particularly in the “Disadvantaged communities” section.
Zakar et al. (15)	15	High	A mix of purposive and snowball sampling. This is a bit unusual, but justifiable since the objective was to capture participants from multiple cities.	Not applicable	Theme identifications were performed by multiple analysts, and difference in coding/interpretation were resolved through discussion → considered to follow the recommended practice.	Pure qualitative study, without statistical analysis	All of the themes are supported by actual quotes by the participants. There are inconsistencies in quote attribution, however. For example, several quotes only had “one participant said” but some have age, gender and occupation. This reduces the trustworthiness of the quotes.	The themes and recommendation being discussed were based on the interview results

## 3. Results

### 3.1. General population

Studies on stigma and COVID-19 have revealed that it affects not only the COVID-19 patients and survivors, but the general population as well. Stigma resulted in delayed health-seeking behavior and preventive efforts (8), with studies identifying anticipated stigma and stereotypes as barriers to COVID-19 testing (9). Herawati et al. (10) conducted a cross-sectional study with a total sample size of 451 respondents consisting of students in the field of health studies and religious studies in West Java, Indonesia. Data was collected using an online questionnaire, which consisted of variables of anxiety, stigma, economic conditions, religiosity, and prevention of COVID-19. The results showed that the most significant influential variable on COVID-19 preventive efforts was the stigma with an Odds Ratio (OR) of 2.256, that is, individuals who experienced high stigma had twice the risk of making a low preventive effort compared to individuals who experienced a low stigma. This study also observed that due to COVID-19, 62% of respondents reported a decrease in income. However, no relationship was seen between levels of religiosity and COVID-19 preventive efforts (10). Another study surveyed 157 German participants regarding their intention to comply with government issued behavioral recommendations. They found that young males were most likely to display low compliance, stressing the need for selective health promotion efforts. They also observed that public stigma had a positive association with compliance (11).

Certain variables are associated with a greater likelihood of one developing a stigma toward COVID-19, including lack of knowledge about the disease, self-employment, financial constraints, and pre-existing depression (9, 12). A cross-sectional survey by Jiang et al. (4) conducted in 31 provinces in China covering 5,039 respondents identified various factors related to COVID-19 stigma. This study indicated that participants over the age of 40, who were ethnic minorities and who felt it was difficult to find and understand information were more likely to stigmatize COVID-19 patients (4).

Apart from stigma, COVID-19 transmission led to significant economic, political, social, legal, and cultural challenges. Multiple studies have verified that COVID-19-related lockdown strategies have increased unemployment, leading to poverty, hunger, and restricted access to healthcare (13). This widespread economic instability has led to psycho-social and socioeconomic insecurity (14).

Zakar et al. (15) conducted a qualitative study based on 34 telephone or online in-depth interviews with participants from diverse age groups in the Punjab province of Pakistan. A semi-structured interview guide was used for data analysis, which included questions regarding problems the study participants experienced to observe public health measures in their households, in their neighborhoods, and social spaces. Probing questions were added to ask about social and cultural factors and challenges they faced in implementing COVID-19 protective measures. The study showed that apart from people’s poor understanding of the virus and the need for containment measures, false and misleading information about the coronavirus has significant consequences on containing the virus. This study also shed some light on religious practices or beliefs as another obstacle in flattening the COVID-19 curve. This has been observed in developing countries and several African communities (15).

### 3.2. COVID-19 patients and survivors

There are various types of stigma that COVID-19 patients and survivors have to deal with. Social rejection and labeling were among the most notable domains of stigma experienced by these people (16). Lohiniva et al., indicated that respondents did not feel a sense of closure after their isolation and quarantine had ended because of perceived stigma, self-stigma, and worry that they could still infect people around them (8). In addition to this; perceived external stigma, internalized stigma, disclosure stigma, and financial insecurity were commonly reported by the COVID-19 affected population. Yuan et al., conducted a cross-sectional study comprising 154 COVID-19 survivors and 194 healthy controls. COVID-19-related stigma were measured by the Social Impact Scale (SIS) and stigma differences between the two groups were compared. The study results found that COVID-19 survivors experience significantly more overall stigma and heightened stigma in domains of social rejection, financial insecurity, internalized shame, and social isolation compared with healthy controls. Another interesting finding of the study was that married people reported higher levels of stigma than those who were unmarried, which is consistent with earlier reports (12).

The magnitude of the impact of the pandemic was also influenced by the socio-economic and ethnocultural differences among the population. Miconi et al., performed an online survey to investigate the association of sociocultural characteristics and pandemic-specific risk factors (i.e., exposure to the virus, COVID-19-related discrimination, and stigma) with mental health during the COVID-19 pandemic in a culturally diverse sample of 3,273 adults in Quebec (Canada). The results showed that socioeconomic status (in terms of income and household size) and race/ethnicity were both associated with mental health, beyond the contributions of prior mental health, experiences of discrimination not related to COVID-19, and other sociodemographic variables. In comparison to other sociocultural groups, Black participants reported the worst mental health results when exposed to the virus and/or COVID-19-related discrimination (17).

Fear and stigma that accompany the pandemic have adversely affected the mental health of this subgroup in particular (12, 17). Literature on psychological consequences of stigma on this population reported that higher levels of depression, anxiety, insomnia, and Post Traumatic Stress Disorder (PTSD) were attributed to being the victim of stigma (18). Studies demonstrated that being subjected to stigma also had a detrimental effect on the quality of life of the patients (19).

### 3.3. Others

There were special subgroups of the population that have been overlooked during the pandemic and need special attention to alleviate their fears and concerns. Firstly, the caretakers who are ill informed and overly burdened required additional guidance and support. Secondly, children and teenagers were easy targets of stigma, as they do not have adequate opportunities to discuss their apprehension and gain correct information. Thirdly, when compared to those who tested positive, asymptomatic family members or those who tested negative received less care, while having equally significant concerns (8).

### 3.4. Quality appraisal of included studies

Despite the varying objectives and methodologies of the reviewed studies, four aspects merit being discussed to better inform future investigations of stigma and COVID-19. The four aspects were (1) measurement tools, (2) sample selection, (3) use of online platforms, and (4) design of qualitative studies. First, regarding the measurement tools, investigators have used stigma-related questionnaires originally developed for PTSD, AIDS, Ebola, mental health, or general health and Quality-of-Life questionnaires. Although several adaptations and pre-testing have been performed, we should caution that several aspects of the questionnaires might not be appropriate in the context of COVID-19. For example, the behaviors related to the disease transmissions were different for AIDS and COVID-19 (unprotected intercourse vs. not wearing a mask), and the barriers related to their protection efforts are different as well (buying condoms vs. masks). The use of MERS questionnaire by Kang et al. (18) might be more appropriate, given the relative similarity of transmission methods between MERS and SARS-CoV-2 viruses. Ideally, however, we should start developing tools that specifically explore COVID-19-related stigma.

Second, regarding sample selection, in all of the studies reviewed, investigators provided reasonable rationales and criteria for sample selection based on the objectives of the studies. However, we noted one inconsistency in the Herawati et al.’s (10) study, where age above 30 years was an exclusion criterion, but the range of participants was reported as 17–49 years without any explanation (we have sought further clarification from the authors). Despite the small number of participants in the case where consecutive sampling was performed (particularly for qualitative studies), the stopping criteria used were data saturation which we deemed as appropriate considering the objectives.

Third, given the COVID-19 restrictions on face-to-face surveys and interviews, several studies have relied on the use of online survey platforms. However, we found that except for Earnshaw et al. (9) most studies reported insufficient details on the timely completion of the surveys, data quality checks, and duplicate entry prevention measures. These were recommended measures to be taken to minimize bias in online surveys. For example, IP address checks (Internet Protocol address—the label connected to a computer network that uses the Internet) would deter multiple surveys completed by a single person motivated by financial rewards given by the completion of each survey. Similarly, utilizing a platform where durations of surveys were recorded would enable researchers to exclude surveys completed in an unreasonably short time (by automated software’s like Chat GPT or copied and pasted) to weed out bad data submitted.

Fourth, for the studies that have qualitative components in them, we found that the themes or ideas presented by the authors were well-supported by actual quotes from the participants. However, we did not find a study where participants provide corrections on the transcriptions and feedback on the findings. These two important aspects were in line with the consolidated criteria for reporting qualitative studies (COREQ) recommendations by Tong et al. (20), to ensure that the themes reported were in line with what the participants’ thoughts were. We hope that these steps were performed in future investigations.

We also analyzed the studies with some more merits to check the quality. All the included studies have well-defined research questions and objectives. Almost 50% (7) of the included studies have specified and defined the study population. One study (12) reported that the participation rate of eligible participants was at least 50%, others did not report. The studies (8–11, 13, 14) had included the participants from the same population during the defined period with prespecified inclusion and exclusion criteria, uniformly applied to all eligible participants. Only one study (16) reported justification of sample size, power description/variance, and effect estimates.

## 4. Discussion

This paper aimed to examine the psychosocial and cultural issues associated with the COVID-19 pandemic. We attempted to include as many studies as possible that examined stigma, mental health access, cultural barriers, myths, and misinformation associated with the COVID-19 pandemic. COVID-19 has resulted in a high level of stigma, anxiety, public confusion, and fear in the setting of many unknowns surrounding this virus. Stigma is a key component of inequalities, but it has been largely ignored in the debate over COVID-19’s response. Stopping and controlling pandemics, assisting societies in their recovery from pandemics, and achieving equitable development are all hampered by stigma. For example, African Americans are one of the highest-risk categories when it comes to dying from COVID-19, and yet they may be more resistant to being vaccinated. This mistrust toward people in authority can perhaps be traced back to the dark times of slavery, and has been perpetuated by the violence and apathy, exerted on them ever since. The community remembers, and naturally do not trust any governmental interference in their health, even if it is likely benevolent. The most prominent example of this is the Tuskegee syphilis experiment participants, who were unethically studied in the 1930s through the 1970s by the government in a study on syphilis (21). African American men infected with syphilis were deliberately left untreated to understand what happened to them over time (even when treatment became available and was being prescribed to others). This mistrust also led many African Americans men to fear that racial profiling and police harassment will worsen if they will wear masks (22). Those who choose not to wear a mask in order to not be perceived negatively in the eyes of a racist society can become potential vectors. The failure of the public healthcare system to take these factors into account presents a lose-lose situation.

Common miscomprehensions of what viruses and pandemics are and how they spread lead to resistance in people toward what can help; that being social distancing, temporary but aggressive lockdowns, and the stringent use of face masks. When communities blinded by religion or cultural habits do not look through a scientific lens; they may be more likely to overlook disease symptoms. This was the case in the early days of COVID-19, whose symptoms were deemed akin to those of the common cold or flu (23). An ongoing conflict between culturally appropriate and medically appropriate pandemic practices can cause psycho-cultural trauma. This can hinder the ability of individuals to cope with stressors and can make recovery difficult (24). Collective trauma (trauma that negatively impacts entire societies) leaves even longer-lasting effects with even fewer outlets available to manage despair or allow for cathartic emotional release (i.e., venting). The potential for mass trauma makes it crucial for governmental organizations and public health officials to work together to provide outreach and actively disseminate beneficial coping information.

This “us” vs. “them” dynamic indicates a way in which disease stigma can be viewed as a proxy for other types of fears like xenophobia. The pandemic risk associated with SARS-CoV-2 infection led to the realization of how stigma and discrimination can remain barriers to care for people suspected of being infected; even more if they were frontline healthcare workers or assisting them (2). Recognizing disease stigma; exploring it, and not simply blaming the ignorance of others, can give us insight how these attitudes are formed and how we can disband them. One should keenly reflect on historical evidence to determine what interventions against stigma surrounding infectious diseases has succeeded in the past to determine what may work for COVID-19 pandemic (2).

It is essential to address COVID-19-related stigma to contain the spread of the virus. To overcome this stigma, several agencies, scientific publications, and experts have issued recommendations and taken relevant initiatives. These recommendations have emphasized the usage of inclusive language when talking about the disease, avoiding the spread of misinformation and rumors, being thoughtful and supportive while communicating, and propagating clear, actionable information to support communities affected by this outbreak.

International public health organizations tend to view outbreaks through the lens of epidemiology and hard medical facts. Nevertheless, it could be beneficial to add psycho-cultural nuances to rote medical approaches. For example, during the Ebola crisis, the World Health Organization (WHO) was able to successfully flatten the Ebola transmission rate by melding its treatment strategies with the cultural practices, social norms, and beliefs of affected communities (25). Another example is that Japan’s remarkably lower COVID-19 death rates. This was achieved by telling people to avoid the three “Cs”—closed spaces, crowded places, and close-contact settings, rather than solely instructing them to stay at home (26). Their approach also harnessed a long held cultural belief in several Asian countries that people wear facemasks in public during the influenza season with an expectation that it helps prevent infections (27).

Global health organizations should also study how to mitigate cultural taboos as they apply to COVID-19, even if they may not be up to par with the original standard. For instance, if conditions compliant with hospital protocol were followed, the risk of infectious spread would be lowered, and more people could be permitted to attend gatherings like funerals. To this effect, immediate family members, in full personal protective equipment, could be allowed to visit their passing loved ones, say their goodbyes, and perform their final rites. Such solutions are neither meant to be compromises, nor supposed to be perfect. Rather, they sensitively cater to the needs of different cultures so that individuals can engage in at least some form of their cultural practices or religious rituals while also adhering to health prescriptions for the greater good. Only with innovation, cultural sensitivity, and perseverance can these divides be healed, and the mental trauma induced by COVID-19 be diminished. The current pandemic presents us with an opportunity to introspect, educate ourselves, and understand narratives that have been previously misunderstood or underrepresented.

Social isolation, anxiety, fear of contagion, uncertainty, chronic stress, and financial difficulties may lead to the development or exacerbation of stress-related disorders and suicidality in vulnerable populations including individuals with pre-existing psychiatric disorders, low-resilient persons, individuals who reside in high COVID-19 prevalence areas and people who have a family member or a friend who has died of COVID-19 (28). Social disengagement played an important role in the elevated suicide rate during the 2003 SARS epidemic in Hong Kong (29). It is concerning from the suicide prevention perspective that social isolation is the most crucial public health strategy for the COVID-19 pandemic. COVID-19 survivors, particularly those who experienced severe symptoms are at elevated risk of suicide (30). Stressful events such as learning about the diagnosis of COVID-19, fear of spreading the infection to others, symptoms of the illness, hospitalization, especially admission to an intensive care unit, and loss of income may lead to the emergence of anxiety, depression, and PTSD (30, 31). Suicide prevention in the COVID-19 era is an important and difficult issue and policymakers should create well-defined guidelines to help clinician manage such cases.

During the COVID-19 pandemic, the personal liberties embraced by individuals in the western world made it difficult for public health officials and local and federal governments to contain the spread of this disease (32). United States (U.S.) citizens have defied and protested their state’s stay-at-home orders out of the belief that public health interventions “have gone too far” and violated their rights. This was exacerbated by misinformation disseminated *via* media (5). For example, some Michigan citizens belonging to working-class population believed that local leaders were “out to get them” by disrupting their local economy, thereby stripping them of their livelihoods and liberty. In April 2020, protesters gathered outside Michigan’s capitol building in Lansing, in large crowds without masks and brandished flags, banners and guns outside and inside the building and at the gates of health facilities (32). They demanded that their politicians repeal the stay-at-home order. Their rally defied social distancing and prevented healthcare workers (some of whom passively counter-protested) from getting to work on time. This situation put the Libertarian protesters, their healthcare worker counter-protesters, their patients, and frontline peers at a greater risk of infection (33). This resistance to quarantine and isolation measures in the U.S. contributed to the ineffective mitigation of the COVID-19 pandemic. Numerous studies pointed to the effectiveness of masks and social distancing, thus identifying a lack of adherence to isolation and masking policies being a major factor in intensifying the spread and impact of COVID-19 (34, 35). People’s intention to comply with recommendations was also found to be an important factor in the successful containment of the COVID-19 pandemic (36).

Some of these same obstacles were seen when the virus broke out in a less liberal society, but not to the same degree (35). In China people have less private control over their lives and the government controls every psycho-cultural echelon of society, including the healthcare system. China saw fewer protests and petitioning as people may have felt that it will be futile, and there were no citizens occupying government buildings or major hospitals. Not surprisingly, when China imposed a lockdown due to COVID-19, the government’s policies were strictly followed. Following the initial outbreak in Wuhan, China implemented “harsh” containment measures, which resulted in a 90 percent reduction in COVID-19 cases in 2 months (37). The doctors who did speak out in the early months of 2020 were summarily suppressed along with their social media accounts, as information spread is tightly regulated (38). Social distancing and the wearing of face masks presented a cultural shock to many communities. Enforcing these guidelines compromised several communal religious events, ceremonies, rituals, and burials. In many communities, people value their rituals and faith as above science and the advice of health experts, and find themselves paralyzed to see their practices being disrupted.

Policymakers and healthcare workers should collaborate in efforts to disseminate factually correct knowledge regarding COVID-19. It is important for governments to consider applying previously established evidence-based stigma reduction strategies to the current pandemic (39), the public should exercise caution in its consumption and response to COVID-19-related media content. This may help minimize the associated anxiety and decrease the likelihood of succumbing to misinformation and conspiracies, hence contributing to improved COVID-19 preventative efforts. It would be beneficial to enable support groups (*via* the internet if movement restrictions are in place), particularly for COVID-19 affected patients during their confinement and to make mental health services more accessible. All household members of affected patients should receive age appropriate education pertaining to the disease and measures to manage it. Psychological assistance should be specially provided to those traumatized by their experiences, including healthcare workers, caregivers, and victims of COVID-19 hate crimes. Access to healthcare, education, and sustained connectivity to the outside world can help re-establish a sense of “normalcy,” and therefore, be crucial to the success of containment facilities during an outbreak.

We should also acknowledge the role that the COVID-19 pandemic has played in exacerbating preexisting social and ethnic-racial inequalities. Policies should focus on improving social inclusion, reducing the discrimination of minority groups, and ensuring that mental health services are accessible and appropriate to the needs of racial, ethnic, and religious minorities, both during and after the pandemic.

Limitations of our scoping review include the heterogeneity in sample sizes (30–5,039) and the type of study conducted. Another limitation of our scoping review is the rapidly evolving nature of the COVID-19 pandemic, which may lead to outdated information as new research and data become available. Additionally, the review may not cover all relevant aspects of psychosocial and cultural factors due to the vastness of the subject matter and potential language barriers in accessing international sources.

## 5. Conclusion

The COVID-19 era requires public health officials and government leaders to consider a broad range of cultural and religious involvement when devising a plan to curb the spread of this virulent disease. Aligning healthcare practices with cultural sensitivities is more likely to help control pandemics like COVID-19. Although science-based approaches have been successful in decreasing the spread of COVID-19, the secondary psycho-cultural effects of the virus on minority communities like Asians and Asian Americans (due to the virus’s perceived origins in China) and African American communities (given the racial bias and discrimination prior to the pandemic) remain significant and could persist for years to come. Incorporating more psycho-culturally aware healthcare practices and policies could be advantageous in managing the pandemic and its potentially multilayered aftermath.

## Author contributions

SA and SB determined study design, contributed to developing the original protocol, and revised the manuscript. SA contributed to data analysis and interpretation of results. LJ contributed to the original screening of papers, data extraction, writing the first draft of the manuscript, contributed to the introduction, results, discussion section, and references. LJ and SA reviewed the manuscript. SB, LJ, and SA contributed to writing the manuscript, analyzing data, interpreting data, and writing the introduction. HB, SS, and NN contributed to writing the introduction, discussion, and literature search. AM and AZ contributed to analyzing data, interpreting data, and writing the discussion. NM, MJ, and RA contributed to the literature search, writing some sections of manuscript, and making tables. VS contributed to the literature search and writing some sections of manuscript. All authors contributed significantly to the research, have knowledge of the topic, made a substantial contribution to writing, agreed to the final version of the manuscript, and met all ethical requirements.
